# Outcomes of a model for re-testing HIV-negative index contacts in Sedibeng, South Africa

**DOI:** 10.4102/sajhivmed.v24i1.1482

**Published:** 2023-05-29

**Authors:** Ditebogo L. Phiri, Kate Rees, Natasha Davies

**Affiliations:** 1 HIV Testing Services, Faculty of Public Health, Johannesburg, South Africa; 2 Anova Health Institute, Johannesburg, South Africa; 3 Department of Community Health, School of Public Health, University of the Witwatersrand, Johannesburg, South Africa; 4 Department of Clinical Care, Faculty of Public Health, Anova Health Institute, Johannesburg, South Africa

**Keywords:** index contacts testing, routine data, HIV case-finding, seroconversion, sero-different couples, pre-exposure prophylaxis, REDCAP, U=U

## Abstract

**Background:**

Index contact testing is an HIV case-finding approach that elicits sexual or needle-sharing partners, as well as biological children, of people living with HIV (PLHIV) and offers them HIV testing services.

**Objectives:**

We aim to describe the results of an innovative project in Sedibeng District that expanded index testing by retesting previously negative contacts and incorporating status-neutral testing.

**Method:**

We used registers to identify people who previously tested HIV-negative through index testing from March 2019 to September 2021. The individuals were telephonically traced and offered HIV retesting. Data were collected on a weekly basis using REDCap^®^. We monitored the number of individuals called, those who came back for retest, and their HIV results.

**Results:**

Fifteen counsellors contacted 968 people over 12 months. Forty-eight percent (462 out of 968) of those called returned for testing. Of those, 121 (26%) tested positive. Overall, 66 out of 276 (24%) men with HIV and 55 out of 186 (30%) women with HIV were identified and linked to antiretroviral treatment (ART). Fifty-seven percent (194 out of 341) of clients who tested HIV-negative were offered, and 124 out of 194 (64%) initiated, pre-exposure prophylaxis (PrEP). All individuals who retested HIV-positive had a new diagnosis; none reported having had another positive test result between the original negative and the positive retest.

**Conclusion:**

Revisiting index clients with a previous negative HIV test result is worthwhile, creating an opportunity to identify undiagnosed PLHIV and high-risk people for PrEP. The high positivity rate highlights the importance of providing a sero-neutral approach to HIV testing, including integrating prevention messaging and linkage to PrEP services.

**What this study adds:** This study has found high positivity rates in people who have recently tested for HIV and provides evidence for the importance of integrating prevention messaging and services into HIV testing.

## Introduction

The HIV epidemic remains an important challenge for health systems. Index contact testing is an HIV case-finding approach that focuses on eliciting sexual or needle-sharing partners, as well as biological children, of people living with HIV (PLHIV) and offering them HIV testing services (HTS). It aims to identify people at high-risk of HIV for testing and initiate them on antiretroviral treatment (ART) as soon as possible. Index testing has been adopted by the South African Department of Health (DoH)^1^ and is a United States President’s Emergency Plan for AIDS Relief (PEPFAR) priority.^2^ Index testing is designed to support achievement of the Joint United Nations Programme on HIV/AIDS (UNAIDS) 95/95/95 targets, which aims for 95% of the country’s population living with HIV being aware of their HIV-positive status, 95% of those who have been diagnosed being on ART and 95% of those on ART being virally suppressed.^2^ The 95/95/95 targets were identified to end the HIV epidemic and minimise HIV-related morbidity and mortality. Within this context, index testing is a voluntary service, offered to PLHIV by healthcare providers. Index testing messaging to encourage uptake by PLHIV has been framed to present an opportunity to access disclosure assistance and HTS for their partners and children. For HIV programmes, index testing has been identified as an important approach for case-finding, improving positivity rates, and reaching hard-to-find PLHIV for testing, especially men.^3,4,5^ The advantages of such a targeted approach are particularly important in South Africa, where the number of PLHIV who are unaware of their status has decreased to less than 7% and identifying those who still need to be diagnosed has become more challenging.^2^ ART coverage, however, remains suboptimal^6^ and index testing can also help to identify partners or children who are living with HIV and are currently disengaged from care or were not linked after diagnosis.^2^

Sero-different couples, where one partner has HIV and the other does not, are common in sub-Saharan Africa,^7,8^ contributing to continuing high population transmission rates.^9^ Although it is now known that HIV cannot be transmitted once viral suppression on sustained ART has been attained, referred to as U=U (undetectable is equal to untransmittable), the protection of the HIV-uninfected partner is only conferred once the partner living with HIV is consistently adherent to ART, and has a viral load below 200 copies/mL.^10,11,12,13^ Index testing is most often offered to people who are newly diagnosed with HIV, those who are not virally suppressed, and those who are re-engaging in care after treatment interruption. Thus, contacts of individuals being offered index testing are often not able to benefit from the protection of U=U and remain at higher risk for HIV acquisition, at least until their partner is stable and adherent on ART. Sero-different couples therefore remain an important target population for HIV prevention initiatives.

In addition to its importance in case-finding, index testing provides an opportunity for targeted HIV prevention, by providing a point of entry into health services for the negative partner in sero-different couples, who is at substantial risk of HIV acquisition while their partner is becoming virally suppressed on ART.^7^ The scale up of oral pre-exposure prophylaxis (PrEP) in South African health services^14^ provides an opportunity to expand index testing to incorporate the principle of status-neutral testing and offer PrEP to negative partners in sero-different couples.^15,16^ Sedibeng facilities started rolling out PrEP in 2020; however, we only started offering PrEP midway during the project, as we continue to integrate prevention services into HTS.

Anova Health Institute (Anova) is the PEPFAR District Support Partner in Sedibeng District, South Africa. In partnership with Sedibeng Health District, alongside standard index testing strategies, we implemented an innovative project aiming to ensure that adults known to be in sero-different relationships are offered retesting for HIV and linked to HIV prevention services, including PrEP. We contacted people who had previously tested negative through index testing services and asked them to return for repeat testing. This article describes the uptake and test results of the index contact retesting approach.

## Methodology

### Setting

The study took place in Sedibeng Health District in Gauteng province, South Africa. The Naomi Model estimates that 110 000 PLHIV resided in Sedibeng in 2021, 40% of whom were men.^17^ The majority (94%) of PLHIV were estimated to know their status, and 89% of those with known status were on ART. Of the PLHIV not on ART, 52% were men.^17^ The Naomi Model is used by the DoH and PEPFAR for planning and programme monitoring in South Africa. It uses programme and survey data to estimate key HIV indicators at district level.^18^

### Programme description

Anova supports the standard DoH HIV index testing approach utilised across all facilities in Sedibeng District. As part of a quality improvement intervention, the project team developed an innovative element by revisiting individuals who had previously tested HIV-negative as part of routine index testing services. The team undertook a standardised quality improvement project (QIP) approach, utilising the Plan-Do-Study-Act (PDSA) cycle model to implement this new approach and analyse the outcomes and impact.^19^ The QIP was implemented in 22 primary healthcare facilities across Sedibeng District.

Following development of the QIP, 15 HIV testing team counsellors identified individuals who previously tested HIV-negative through index contact testing from March 2019 to September 2021. The implementation of the project started in October 2021, and we report data through September 2022. These individuals were identified by reviewing the index testing registers completed during the time of their last HIV test. We targeted individuals who were in sero-different relationships (index client tested positive and elicited partner tested negative), grouped them according to the date of their last test, telephonically traced them and invited them to facilities for repeat HIV testing through Anova’s HIV testing counsellors. The individuals who retested HIV-positive were linked to and initiated on ART on the day of their new positive test result while those who tested HIV-negative were offered and linked to prevention services (PrEP, voluntary medical male circumcision, condom provision). The counsellors offered and performed HIV testing according to the National HTS algorithm 2016 revised version, which includes a second confirmatory test should the first result be positive.^20^

As the project progressed, we developed scripts for common situations encountered by counsellors when telephonically recalling clients (e.g. no longer with the same partner). We also shifted the messaging towards a sero-neutral testing approach wherein individuals were encouraged to test to facilitate access to PrEP to prevent HIV acquisition, rather than solely testing to become aware of a positive HIV status. For example, the script for initiating the conversation (after introductions) ran: ‘I am just calling to let you know that we now have PrEP in our facility’ and then provided an explanation about what PrEP is, and issued the invitation for a HIV test: ‘If you are interested then the first step is to come in to meet with me so you can have another HIV test to confirm that you are still HIV-negative’.

The scripts are included as Online Appendix 1. Confidentiality was maintained throughout the implementation of the project, no data were shared beyond the project team and identifying information was removed during analysis. All our testers were trained on how to provide index testing in a safe and ethical manner according to South African guidelines and DoH index testing standard operating procedures SOP000108/2020.

### Data collection and analysis

**FIGURE 1:**
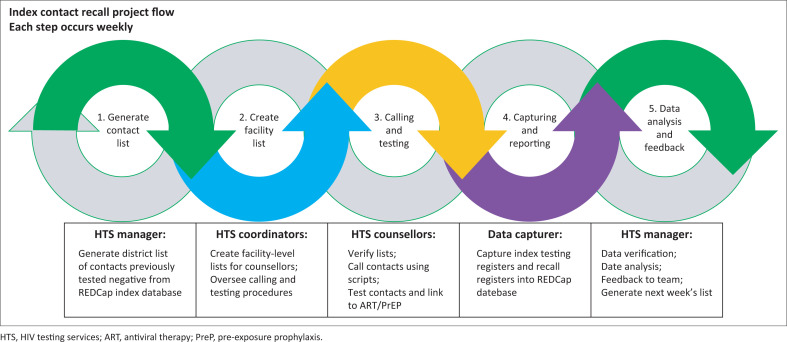
Index contact recall weekly process flow, Sedibeng District.

Data were collected on a weekly basis using a REDCap database (see Figure 1).^21,22^ Anova uses REDCap to capture routine programme data. We collected demographic information including age and gender, as well as the date of the repeat test and the repeat test results. Three telephonic attempts were made at different times to reach the index contacts before designating ‘unreachable’ as an outcome.

We present descriptive statistics for all contacts 15 years and older, including the number of contacts recalled, the rate of return, positivity rate, and PrEP offer and initiation rates. Results are presented as frequencies and proportions. The number of patients who declined repeat HIV testing is reported, including the reasons for declining repeat testing. To investigate associations between age or gender and testing, positivity, PrEP offer, and PrEP enrolment rates, we used the chi-squared test for difference between proportions.

### Ethical considerations

Ethical clearance to conduct this study was obtained from the Human Sciences Research Council (No. REC 3/22/08/18). Because this QIP involved only the use of anonymised aggregated programme data, no informed consent was obtained.

## Results

Over the 12-month period, 968 people were contacted by the HTS counsellors. Sixty percent (*n* = 577) were male, 15% (*n* = 142) were between 15 and 24 years old, 66% (*n* = 637) were between 25 and 44 years old and 8% (*n* = 74) were 50 years or older. Just over one-third of people contacted (*n* = 362) were unreachable on three attempts using the contact details on record. Forty-eight percent (*n* = 462 out of 968) of those called returned to the facility for testing (Table 1), and the remaining 144 were reached telephonically but declined the offer to retest. Testing rates were the same in women and men at 48% (*P* = 0.936). Since a higher number of the contacts were men, we reached more men for testing (*n* = 276), compared to women (*n* = 186). People 50 years and older were more likely to return for testing than those 25–49 years old (61% vs 47%, *P* = 0.024). Positivity was higher in women compared to men, 30% versus 24%, although this was not statistically significant (*P* = 0.175). Positivity was higher in those older than 25 years (15–24 years: 7%, 25–49 years: 48%, 50 years and older: 42%).

**TABLE 1:** Index testing recalls in Sedibeng District, October 2021 to September 2022.

Index clients	Total		Female		Male		*P* *	15–24 years		25–49 years		*P* **	≥ 50 years		*P* ***
	*n*	%	*n*	%	*n*	%		*n*	%	*n*	%		*n*	%	
Recalled	968	100	391	40	577	60	-	142	15	725	75	-	74	8	-
Tested	462	48	186	48	276	48	0.936	68	48	341	47	0.852	45	61	0.024
Tested positive	121	26	55	30	66	24	0.175	5	7	96	28	< 0.01	19	42	0.052
Tested negative	341	74	131	70	210	75	-	63	93	245	72	-	26	58	-
Offered PrEP	194	57	72	55	122	58	0.570	33	52	142	142	0.425	16	62	0.725
Enrolled on PrEP	124	64	44	61	80	66	0.532	16	48	95	67	0.048	10	63	0.724

Note: 27 records missing data on age.

PrEP, pre-exposure prophylaxis.

* , Chi-squared test women versus men;

** , Chi-squared test 15–24 years versus 25–49 years;

*** , Chi-squared test 24–49 years versus 50 years and older.

Overall, from the 968 individuals identified from the index registers for recall, 12.5% were newly diagnosed with HIV within a maximum interval of 2.5 years (March 2019 – September 2022) between initial negative and subsequent positive test results. All those identified as PLHIV were newly diagnosed with HIV at retest, none of them having reported any other positive test in the interim between their original index testing encounter and this retesting intervention. All 121 of these individuals were successfully linked to ART following their diagnosis.

Of the 341 clients who retested HIV-negative, 194 clients were offered PrEP (57%) and 124 enrolled on PrEP (64% of those offered). Pre-exposure prophylaxis offer rates were similar in age and gender groups, but PrEP uptake was lower for young people aged 15–24 years compared to people 25–49 years old (48% vs 67%, *P* = 0.048).

For the 144 clients that declined the offer of retesting, the following reasons were given: not ready to test again (17%, *n* = 24), no longer with the same partner (46%, *n* = 65), moved to another province (38%, *n* = 55).

## Discussion

We have described an innovative QIP designed to strengthen outcomes of HTS services through recalling previously HIV-negative index contacts for retesting at least 1 year after their initial contact testing engagement. We found high positivity rates, with just over one-quarter of previously HIV-negative individuals retesting HIV-positive, implying high rates of seroconversion in this population. This project had three important programmatic outcomes: identifying high-risk people to offer PrEP, identifying HIV quickly in those who had seroconverted since their last HIV test, and reaching a high proportion of men.

The project has highlighted high infection rates in sero-different relationships, despite the positive partners having accessed ART by the time of initial index testing, and the negative partner accessing HIV testing. It is possible that some of the individuals who retested positive were in the window period during their first test, and either were unaware of the need to retest or did not attend as recommended after 3 months. In both cases, recalling index contacts for retesting provides a way to keep interacting with people at high-risk. It also points to missed opportunities for linkage to HIV prevention services during HTS interactions where the clients test HIV-negative. Although the project initially started with just offering HIV retesting, the need to integrate prevention services, particularly PrEP, quickly became clear, to support the client who tested negative to remain so. Oral PrEP has been available in public health facilities in Sedibeng since 2020. We only started offering PrEP midway during the project period but found that more than half of those offered PrEP took up the offer and initiated PrEP, including in men, although adolescents and young people had substantially poorer PrEP uptake. As well as providing PrEP services, education and awareness are needed for both service providers and users to ensure that prevention messages are structured in ways that are accessible and easily understood. Greater effort is needed to strengthen the offer of PrEP to as many as possible of those testing HIV-negative through index or repeat HIV testing and to understand the reasons clients decline PrEP despite being identified as belonging to a high-risk group. Couple-focussed approaches would enable follow-up of the positive partner to ensure adherence on ART and support couples to reach a situation where U=U can be in place. A greater focus on a sero-neutral approach to HIV testing may help to normalise access to, and uptake of, HIV prevention services as well as strengthening the integration of PrEP offer into index testing and other HTS modalities.

A similar approach was used in Uganda, identifying HIV-negative members of sero-different couples, and offering them PrEP,^23^ although they integrated the service into ART clinics. The trial was successful in reaching a high proportion of people in sero-different relationships.^23^ The Sedibeng HIV testing team used HTS as the entry point to reach a high number of individuals to be enrolled on PrEP, which we show is another useful approach and could be complementary to offering PrEP through ART sites.

In sub-Saharan Africa, there is generally lower awareness of HIV status in men,^24^ and men are often diagnosed late.^25^ Previous studies have shown that men who are unaware of their HIV status have often tested in the past, but do not test frequently enough.^26^ We identified 66 men living with HIV at a 24% positivity rate. Close to half (49%) of the men called returned for an HIV test, and more than half (53%) of all PLHIV identified were men. Although testing rates were similar in men and women, a greater proportion of the original index patients were women, and therefore more men were contacted as partners. Because this modality used telephonic outreach, it may have been successful in reaching men because it doesn’t rely on them already being present in the facility and accessing opportunistic offer of testing once already there. This approach shows promise as a way of improving appropriate repeat testing in men, a priority population in South Africa.

HIV self-testing has been implemented in several settings, including in South Africa, and has been shown to increase acceptability of testing,^27^ including for men.^28^ The approach has also been used successfully to facilitate partner testing.^29,30^ The high positivity noted in this project highlights opportunities to use self-testing in HTS programmes. For example, distributing a self-test, with advice about when to do it, to any person who tests HIV-negative, could support testing after the window period. The return rate of below 50% also requires attention: HIV self-testing services may support greater testing uptake by increasing convenience. In addition, to improve return rates, there is a need to continuously strengthen the process of recalling, including updating contact details of index contacts. Existing stationery should be strengthened to allow for this. A strong emphasis should be placed on communicating the availability and benefits of PrEP, and other prevention services, when people test negative.

It is important for the authors to acknowledge that this project took place during the course of the coronavirus disease 2019 (COVID-19) pandemic, including from the time of South Africa’s first extensive lockdown in March 2020 up until all restrictions were lifted in June 2022. Therefore, it is difficult to assess how much COVID-19 restrictions may have impacted both testing and seroconversion rates within the population reached during the project. COVID-19 drastically impacted healthcare access in South Africa, through myriad mechanisms including health facility closures, limited access to transport, altered health-seeking behaviour and disrupted access to ART for people enrolled in the ART programme.^31,32,33^ It is quite possible that some of the original index clients did not attain viral suppression as quickly or successfully because of COVID-19 resulting in ongoing, higher risks of transmission to their partners. Additionally, their HIV-negative partners may have been less able to access retesting and prevention services in a timely manner. This project is now being rolled out across other Anova-supported districts and we continue to analyse outcomes, including seroconversion rates, beyond the COVID-19 pandemic era.

### Limitations

This project was not designed as a research study, and this analysis uses routine programme data only. There are data quality challenges when using routine data (including missing data and capturing errors), but an added advantage in knowing the approach can be used in a real-world setting. We did not track testing in individuals longitudinally outside of this project, so we are unable to say whether people tested negative during the original window period, whether they had new partners, or whether their partners attended ART visits. We are also unable to summarise the time period between individual’s HIV tests, and so were unable to calculate incidence rates. We have adapted our data collection to enable this going forward.

## Conclusion

Our project indicates that revisiting previously negative index clients is a worthwhile endeavour, both to newly diagnose PLHIV and to identify high-risk clients to initiate PrEP. Index testing should continue to be used as an effective modality to identify PLHIV not yet aware of their status or not on ART, and interaction with contacts should be ongoing, particularly with men, who remain a priority group. The high positivity rate also highlights the critical importance of integrating prevention messaging and linkage to prevention services, including PrEP, into the offer of index testing. Index testing provider training must include a clear emphasis on linking not only those who test positive to ART, but also those who test negative to prevention services. In Sedibeng, during the period of the QIP, rapid ART initiation and index testing were offered simultaneously to all newly identified individuals with an HIV-positive test. Studies have shown that although same-day initiation may be more effective in supporting linkage to ART, achieving high rates of retention and viral suppression remains challenging.^17^ Therefore, some of the seroconversions seen in this population may have arisen from failure to attain U=U with the partners who were rapidly linked to ART. Prospective research studies are needed to establish how best to provide couples-based services to those identified through index testing to mitigate the risk of ongoing, early transmission while people newly diagnosed are engaged on ART before they attain a U=U status.
